# Associations of Muscle Size and Density With Proximal Femur Bone in a Community Dwelling Older Population

**DOI:** 10.3389/fendo.2020.00503

**Published:** 2020-07-28

**Authors:** Lu Yin, Zhengyang Xu, Ling Wang, Wei Li, Yue Zhao, Yongbin Su, Wei Sun, Yandong Liu, Minghui Yang, Aihong Yu, Glen Mervyn Blake, Xinbao Wu, Annegreet G. Veldhuis-Vlug, Xiaoguang Cheng, Karen Hind, Klaus Engelke

**Affiliations:** ^1^Medical Research & Biometrics Center, National Center for Cardiovascular Diseases, Chinese Academy of Medical Sciences and Peking Union Medical College, Beijing, China; ^2^Department of Radiology, The First Medical Center of Chinese PLA General Hospital, Beijing, China; ^3^Department of Radiology, Beijing Jishuitan Hospital, Beijing, China; ^4^Xinjiekou Community Health Service Center, Beijing, China; ^5^Department of Traumatic Orthopedics, Beijing Jishuitan Hospital, Beijing, China; ^6^School of Biomedical Engineering & Imaging Sciences, King's College London, St Thomas' Hospital, London, United Kingdom; ^7^Division of Endocrinology, Department of Internal Medicine, Center for Bone Quality, Leiden University Medical Center (LUMC), Leiden, Netherlands; ^8^Department of Sport and Exercise Sciences, Durham University, Durham, United Kingdom; ^9^Department of Medicine 3, FAU University Erlangen-Nürnberg and Universitätsklinikum Erlangen, Erlangen, Germany

**Keywords:** muscle cross-sectional area, muscle density, quantitative computed tomography (QCT), proximal femur, cortical bone, trabecular bone

## Abstract

**Background and Purpose:** Muscle weakness and bone fragility are both associated with hip fracture. In general, muscle contractions create forces to the bone, and bone strength adapts to mechanical loading through changes in bone architecture and mass. However, the relationship between impairment of muscle and bone function remain unclear. In particular, the associations of muscle with properties of proximal femur cortical and trabecular bone are still not well understood. The aim of this study was to explore the associations of hip/thigh muscle density (CT attenuation value in Hounsfield units) and size with cortical and trabecular bone mineral density (BMD) of the proximal femur.

**Materials and Methods:** Three-dimensional quantitative computed tomography (QCT) imaging of the lumber, hip and mid-thigh was performed in a total of 301 participants (mean age 68.4 ± 6.1 years, 194 women and 107 men) to derive areal BMD (aBMD) and volumetric BMD (vBMD). Handgrip strength (HGS) and the Timed Up and Go (TUG) test were also performed. From the CT images, cross-sectional area (CSA), and density were determined for the gluteus maximus muscle (G.MaxM), trunk muscle at the vertebrae L2 level, and mid-thigh muscle. Multivariate generalized linear models were applied to assess associations.

**Results:** Total hip (TH) aBMD was associated significantly with G.MaxM CSA (men: *P* = 0.042; women: *P* < 0.001) and density (men: *P* = 0.012; women: *P* = 0.043). In women, 0.035 cm^2^ of mid-thigh CSA (95% CI, 0.014–0.057; *P* = 0.002) increased per SD increase in TH aBMD, but this significance was not observed in men (*P* = 0.095). Trunk muscle density and CSA were not associated with proximal femur BMD. The associations of hip/thigh muscle parameters with femoral neck BMD were weaker than those with trochanter and intertrochanter BMD. Furthermore, compared to muscle density, muscle CSA showed better associations with vBMD. G.MaxM CSA was associated with trochanter (TR) Cort. vBMD in men (β, 19.898; 95% CI, 0.924–38.871; *P* = 0.040) and in women (β, 15.426; 95% CI, 0.893–29.958; *P* = 0.038). Handgrip strength was only associated with TR aBMD (β, 0.038; 95% CI, 0.006–0.070; *P* = 0.019) and intertrochanter aBMD (β, 0.049; 95% CI, 0.009–0.090; *P* = 0.016) in men.

**Conclusions:** We observed positive associations of the gluteus and thigh muscle size with proximal femur volumetric BMD. Specifically, the gluteus maximus muscle CSA was associated with trochanter cortical vBMD in both men and women.

## Introduction

Osteoporosis and sarcopenia are both associated with aging, contributing to an increased risk of fracture (1, 2). Sarcopenia refers to reductions in muscle performance with the loss of skeletal muscle mass, while osteoporosis is characterized by deficits in both trabecular and cortical bone. Although close ties exist between their embryogenesis, growth and aging, the relationship between impairment of muscle and bone function remains unclear. The interactions between bone and muscle are not only based on mechanical loading and physical forces created by muscle contractions, but also via endocrine factors (3, 4).

Lower muscle mass and strength are associated with narrower bones and lower areal bone mineral density (BMD) assessed by dual energy X-ray absorptiometry (DXA) (5–7). Although DXA provides excellent precision and total and appendicular lean mass outcomes, it does not distinguish cortical and trabecular bone, and does not provide imaging-based muscle quality assessments. Quantitative computed tomography (QCT) offers the opportunity to distinguish trabecular from cortical bone (8, 9) and provides anatomical muscle assessment (10) because of the three-dimensional (3D) imaging advantages. Several recent QCT-based studies have shown associations between lower spine volumetric trabecular BMD and poorer muscle quality (11, 12). For the proximal femur bone, higher muscle mass by DXA was associated with femoral neck (FN) cortical BMD in older men (13). Little is known about the correlation between the volumetric proximal femur bone density and anatomic muscle assessments. To the best our knowledge, no study has correlated muscle quantity and quality with 3D integral BMD and properties of proximal femur cortical and trabecular bone. The main aim of this study was to explore associations of muscle size and density with proximal femur volumetric BMD assessed by QCT, using data from the China Action on Spine and Hip Status (CASH) study on healthy men and women aged 50–85 years. We also aimed to explore the associations of muscle strength and physical performance with BMD.

## Materials and Methods

### Study Participants

Three hundred and sixteen community-dwelling subjects of at least 50 years of age and in good health, were recruited between March 2017 and June 2017 from the neighborhoods of Beijing Jishuitan Hospital in Beijing, China, using convenience sampling. Exclusion criteria were an (i) inability to sit and stand independently, (ii) inability to walk with or without an assistive device (only relevant for Timed Up and Go [TUG]), (iii) pain that prevented testing and (iv) stroke, neurologic disorders, metabolic diseases, rheumatic diseases, heart failure, severe chronic obstructive pulmonary disease, coagulation disorders, and other diseases that limit function. The study was approved by the ethics committee of Beijing Jishuitan Hospital. Informed consent was obtained from each participant.

### CT Acquisition

Spiral CT imaging (non-contrast) of the hip was performed for all study participants with a Toshiba Aquilion CT scanner (Toshiba Medical Systems Division, Tokyo, Japan). Scans were acquired in supine position from the top of the acetabulum to 3 cm below the lesser trochanter and included both legs. In addition, CT scans of the lumbar spine including vertebrae L1 – L5 and of a 1 cm thick section of the center of the left thigh were taken. The position of this section was determined from a scout view as the center of the long axis of the femur. Scan parameters for all CT scans were 120 kVp, 125 mAs, 50 cm field of view, 512 × 512 matrix, 1 mm reconstructed slice thickness and a standard reconstruction kernel with filtered back projection. Quality assurance was assured through a standardized scanning protocol, fixed scanner table height, and routine water calibration measurements.

### Muscle Density Assessments

**Figure 1:**
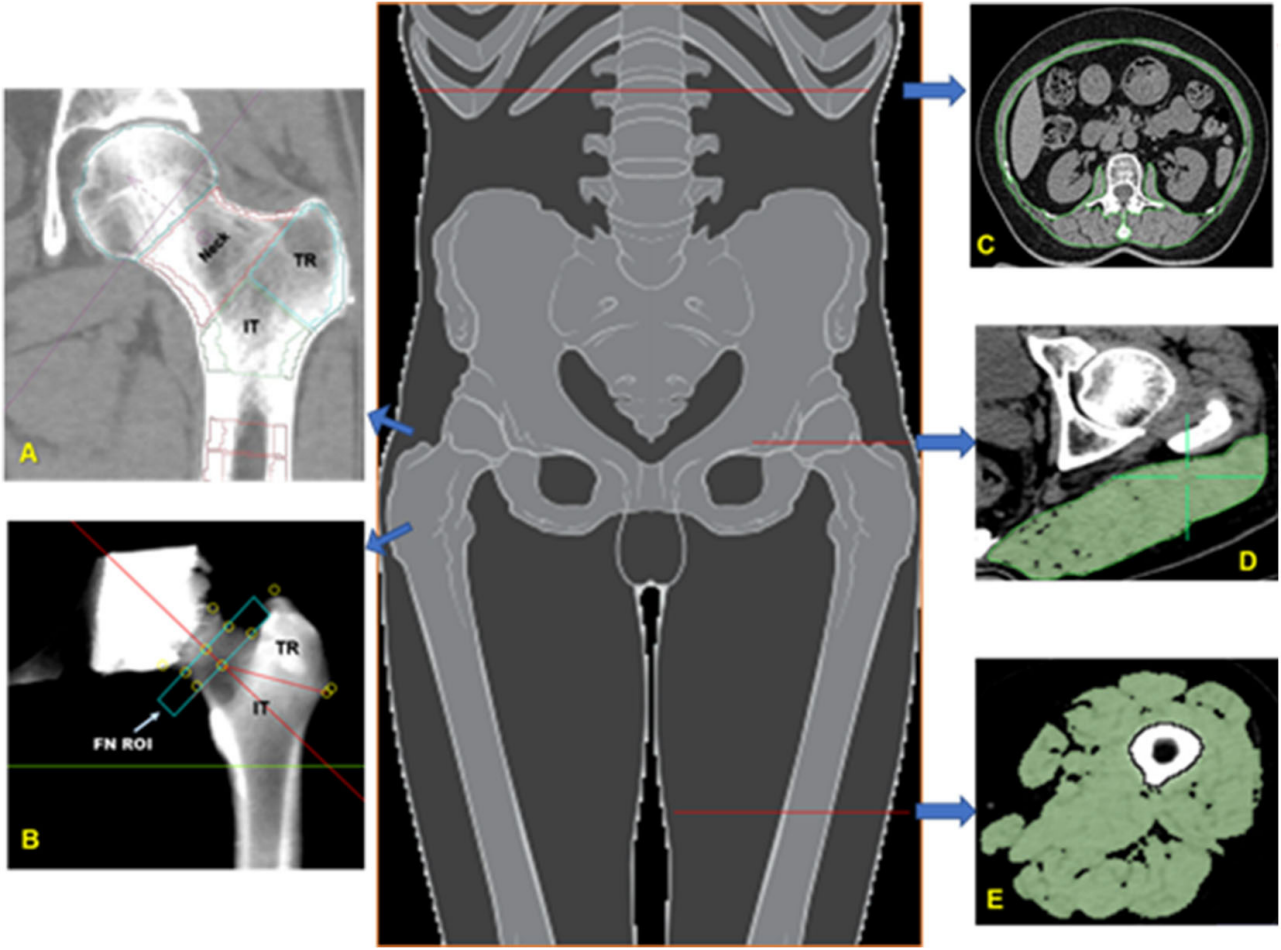
Bone and muscle measurements. Volumes of interest (VOIs) analyzed in the proximal femur by MIAF Femur **(A)** and QCTPro CTXA **(B)**. Measurements of cross-sectional area and mean CT values of the trunk muscle at mid-L2 level **(C)**; Measurement of the left gluteus maximus at the level of the greater trochanter of the femur **(D)**; Measurement of the left mid-thigh muscle group **(E)**.

Cross sectional area (CSA) and density of the following muscle or muscle groups were measured on one slice each. In the hip, the gluteus maximus at the level of the greater trochanter was analyzed. In the trunk the paraspinal muscles (erector spinae and transversospinalis), the posterior abdominal muscles (psoas major and quadratus lumborum), and the anterior abdominal muscles (rectus abdominis, external and internal oblique) were analyzed at the level of L2 (Figure 1). Finally, in the thigh, the ensemble of all muscles (the sartorius, quadriceps, adductors, and hamstrings) was analyzed.

OsiriX software (Lite version 10.0.2, Pixmeo, Geneva, Switzerland) was used for analyses. Muscle segmentation was performed manually using the “pencil” tool to outline muscle contours. Within the resulting muscle regions of interests (ROIs) a threshold of −29 HU was applied to distinguish muscle tissue from fat. All muscle measurements were performed by the same investigator (YZ) who had received training by an expert radiologist (LW) in CT muscle imaging prior to the analysis. For training, a sample of about 20 images had been analyzed together with the expert (LW) prior to the beginning of the measurement study. Excellent intra-observer (intra-class correlation coefficients, ICC 0.932–0.998, *P* < 0.001) and inter-observer (ICC 0.913–0.961, *P* < 0.001) agreements of the muscle measures were found.

### Areal and 3D Bone Mineral Density

Areal BMD (aBMD, g/cm^2^) of the FN, trochanter (TR), intertrochanter (IT), and total hip (TH) was derived from hip CT scans using the CT X-ray absorptiometry technique (CTXA, version 4.2.3, Mindways Inc.). After image segmentation and manipulation of proximal femur rotation, two-dimensional projection images were generated from the 3D CT dataset (Figure 1). Details of the measurement procedure have been described elsewhere (14). The aBMD derived from CTXA is equivalent to DXA and the reproducibility of CTXA measurements was good (15).

The Medical Image Analysis Framework for the Femur (MIAF Femur Version 7.1.0MRH) was used to measure three-dimensional bone parameters of the proximal femur (14). An important advantage of this software is that the three dimensional bone segmentation is combining global and adaptive local thresholds with volume growing and morphological operations (16, 17). Both surfaces are displayed in axial, sagittal, and coronal reformation for operator control and manual editing if necessary. A wide range of 3D editing tools is available (18). The standard set of VOIs obtained by MIAF-Femur are the head, neck, trochanter, intertrochanter, and proximal shaft calculated relative to an anatomic coordinate system (ACS) with its origin centered at the smallest cross section of the neck. The borders between VOIs are determined automatically based on anatomical landmarks and the ACS. Each VOI was separated into integral (Intg), cortical (Cort), and trabecular (Trab) compartments for which vBMD and BMC and volume were determined. The details of proximal femur segmentation and analysis by MIAF Femur have been described previously (14, 19). Precision and accuracy results of MIAF-Femur have been published earlier (14, 20).

### Muscle Strength Assessments

Handgrip strength (HGS) of the dominant hand was measured using a Jamar dynamometer (Jamar®, Los Angeles, CA, USA). Details of the grip strength protocol have been previously reported (21).

### Physical Performance

The TUG test was performed by measuring the time needed to rise from an armchair, walk 3 m on a line drawn on the floor, turn and walk back to the chair to a seated position. Details of TUG test have been previously described (21). The rater who supervised the TUG tests had been trained in detail on how to instruct participants.

### Data Collection

Demographic and anthropometric variables included age, sex, weight, height, hip circumference, and waist circumference. Health-related data included blood pressure, fracture history and the EuroQol five-dimension score (EQ-5D). In this study EQ-5D with 3 levels of severity (EQ-5D-3L) was used. It included five dimensions (mobility, self-care, usual activities, pain or discomfort, anxiety, or depression) divided into three levels of severity (no problems, some problems, severe problems) describing 243 unique health profiles (22). Other health-related data were retrieved from the patient's medical file or from the healthy participants' medical records in Xinjiekou Community Health Service Center.

### Statistical Analyses

All statistical analyses were conducted using SAS 9.4 (SAS Institute Inc, Cary, NC). Means with standard deviation (SD) were calculated for males and females separately. Owing to non-normal distribution from our population, mean differences between both sexes were analyzed using the two-sample Wilcoxon Signed Rank tests, which is one of non-parametric tests, though few data fit a normal distribution. Analyses were stratified by sex due to potential different pathological mechanisms for bone mineral density, muscle structure, and strength. Generalized linear models were fitted for the 14 BMD in top hip, femoral neck, trochanter, and intertrochanter sites. To improve various eight muscle indexes were measured and transferred using sex-specific SD, respectively: (1) G. MaxM area; (2) G. MaxM density; (3) muscle area of middle thigh; (4) muscle density of middle thigh; (5) L2 Trunk muscle area; (6) L2 Trunk muscle density; (7) Grip strength; (8) TUG. Multivariate Generalized linear models were used to assess the above-mentioned associations, adjusted for age, BMI, and EQ-5D.

## Results

### Study Sample Characteristics

Eight participants (from 316) were excluded from the study either because of invalid HGS or TUG measurements. Seven additional participants were excluded because of missing CT scans or because of unacceptable image quality (i.e., artifacts). A total of 301 healthy participants [107 men (age 69.59 ± 6.63 years) and 194 women (age 67.68 ± 5.75 years)] were finally included for analysis, which presented in Table 1. Men had higher muscle area and density in G. MaxM (area, 43.11 vs. 37.29 cm^2^; density, 35.81 vs. 32.14 HU), middle thigh (area, 123.55 vs. 93.13 cm^2^; density, 46.74 vs. 44.18 HU), and L2 Trunk (area, 125.90 vs. 90.29 cm^2^; density, 30.69 vs. 28.00 HU) than women (*P* < 0.01). Men had greater aBMD at five sites including TH aBMD (0.88 vs. 0.75 g/cm^2^), FN aBMD (0.72 vs. 0.65 g/cm^2^), TR aBMD (0.63 vs. 0.53 g/cm^2^), TR Cort. vBMD (451.38 vs. 430.81 mg/cm^3^), and IT aBMD (1.08 vs. 0.92 g/cm^2^), while there was no sex-specific difference for Neck Int. vBMD (280.86 vs. 304.58 mg/cm^3^).

**Table 1:** General characteristics of healthy participants.

**Characteristics (Mean ± SD)**	**Males (*N* = 107)**	**Females (*N* = 194)**	***P*-value^**a**^**
Age (years)	69.59 ± 6.63	67.68 ± 5.75	0.02
Height (cm)	169.71 ± 5.08	158.47 ± 5.33	<0.01
Weight (kg)	72.36 ± 9.40	63.69 ± 8.96	<0.01
BMI (kg/cm^2^)	25.08 ± 2.62	25.33 ± 3.08	0.42
EQ-5D	0.59 ± 0.18	0.58 ± 0.14	0.33
TUG (s)	8.17 ± 1.51	8.28 ± 1.55	0.64
Handgrip (kg)	33.96 ± 7.32	20.97 ± 4.79	<0.01
G.MaxM area (cm^2^)	43.11 ± 7.90	37.29 ± 6.27	<0.01
G.MaxM density (HU)	35.81 ± 6.55	32.14 ± 6.25	<0.01
Muscle area of middle thigh (cm^2^)	123.55 ± 22.24	93.13 ± 14.48	<0.01
Muscle density of middle thigh (HU)	46.74 ± 3.64	44.18 ± 3.84	<0.01
L2 Trunk muscle area (cm^2^)	125.90 ± 19.33	90.29 ± 14.36	<0.01
L2 Trunk muscle density (HU)	30.69 ± 4.48	28.00 ± 4.13	<0.01
Waist circumference (cm)	89.93 ± 8.04	84.78 ± 8.44	<0.01
Hip circumference (cm)	99.28 ± 16.16	97.37 ± 11.83	0.01
Systolic blood pressure (mmHg)	126.55 ± 8.92	126.49 ± 8.50	0.71
Diastolic blood pressure (mmHg)	74.73 ± 5.90	73.76 ± 8.32	0.37
TH aBMD (g/cm^2^)	0.88 ± 0.17	0.75 ± 0.14	<0.01
TH vBMD (mg/cm^3^)	246.92 ± 51.64	255.97 ± 59.21	0.24
FN aBMD (g/cm^2^)	0.72 ± 0.15	0.65 ± 0.12	<0.01
Neck Int.vBMD (mg/cm^3^)	280.86 ± 63.41	304.58 ± 71.26	<0.01
Neck Trab.vBMD (mg/cm^3^)	119.03 ± 46.83	130.26 ± 52.64	0.08
Neck Cort.vBMD (mg/cm^3^)	571.07 ± 84.84	582.01 ± 111.7	0.74
TR aBMD (g/cm^2^)	0.63 ± 0.15	0.53 ± 0.10	<0.01
TR Int.vBMD (mg/cm^3^)	207.04 ± 46.85	204.35 ± 45.02	0.82
TR Trab.vBMD (mg/cm^3^)	84.28 ± 32.57	82.66 ± 31.48	0.91
TR Cort.vBMD (mg/cm^3^)	451.38 ± 77.76	430.81 ± 89.08	0.04
IT aBMD (g/cm^2^)	1.08 ± 0.20	0.92 ± 0.17	<0.01
IT Int.vBMD (mg/cm^3^)	262.35 ± 58.06	275.04 ± 71.63	0.30
IT Trab.vBMD (mg/cm^3^)	103.83 ± 39.73	103.74 ± 45.33	0.93
IT Cort.vBMD (mg/cm^3^)	653.56 ± 106.92	669.00 ± 142.49	0.97
**TH CortThick (mm)**	2.01 ± 0.31	1.87 ± 0.27	<0.01
**Neck CortThick (mm)**	1.93 ± 0.30	1.80 ± 0.26	<0.01
**TR CortThick (mm)**	1.91 ± 0.35	1.77 ± 0.29	<0.01
**IT CortThick (mm)**	2.22 ± 0.37	2.07 ± 0.36	<0.01

*SD, standard deviance; BMI, body mass index; EQ-5D, EuroQol five-dimension score; TUG, Timed Up and Go test; G.MaxM, gluteus maximus muscle; aBMD, areal bone mineral density; vBMD, volumetric bone mineral density; FN, femoral neck; TH, total hip; TR, trochanter; IT, intertrochanter; Int., integral; Trab., trabecular; CortThick, cortical thickness*.

a *P-value was obtained from two-sample wilcoxon Signed Rank tests*.

### Total Hip BMD

**Figure 2:**
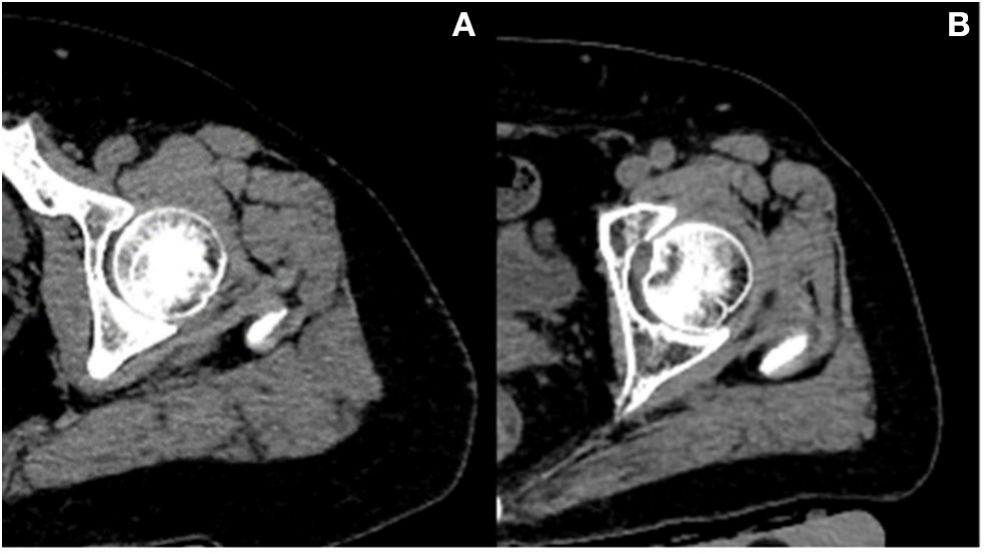
Representative cases with high BMD vs. corresponding muscle CSA/density **(A)** and low BMD vs. corresponding muscle CSA/density **(B)**. Case A vs. case B: both women; age, 61 vs. 62 years old; height, 156 vs. 160 cm; weight, 75 vs. 75 kg; total hip aBMD, 1.00 vs. 0.46 g/cm^2^; G.MaxM area, 53.9 vs. 30.56 cm^2^; G.MaxM density, 36 vs. 33 HU.

Table 2 presents adjusted β and 95% confidence intervals (Cl) for total hip BMD (TH aBMD and TH vBMD) with continuous muscle indexes per sex-specific SD increase in general linear models. After adjustment for additional covariates, TH aBMD are associated significantly with gluteus maximus muscle (G.MaxM) area (men: *P* = 0.042; women: *P* < 0.001) and density (men: *P* = 0.012; women: *P* = 0.043). In women, 0.035 cm^2^ of muscle area of middle thigh (95% CI, 0.014–0.057; *P* = 0.002) and 0.025 HU of muscle density of middle thigh (95% CI, 0.006–0.044; *P* = 0.009) increased per SD increase of TH aBMD, but this significance was not observed in men (area: *P* = 0.095; density: *P* = 0.318). Associations between HGS and TH aBMD were found only in men (β, 0.042; 95% CI, 0.008–0.076, *P* = 0.018), but not in women (*P* = 0.127). Another four BMD indexes were found to be unrelated to THaBMD (*P* > 0.05). TH int.vBMD 10.619 cm^2^ of G.MaxM area was raised per SD increase only in women (95% CI, 1.015–20.224; *P* = 0.030). Figure 2 shows representative cases with high TH aBMD vs. corresponding muscle CSA/density(A) and low TH aBMD vs. corresponding muscle CSA/density(B).

**Table 2:** Adjusted β and 95% CIs for sex-specific SD increase of total hip BMD with various muscle indexes^a^^,^^b^.

**Variables**	**TH aBMD (g/cm**^****2****^**)**		**TH Int.vBMD (mg/cm**^****3****^**)**	
	**β (95% CI)**	***P*-value**	**β (95% CI)**	***P*-value**
**Males**				
G.MaxM area (cm^2^)	0.037 (0.001, 0.072)	0.042	6.243 (−6.371, 18.858)	0.328
G.MaxM density (HU)	0.044 (0.010, 0.077)	0.012	7.707 (−3.938, 19.352)	0.192
Muscle area of middle thigh (cm^2^)	0.030 (−0.005, 0.065)	0.095	−0.656 (−12.897, 11.586)	0.915
Muscle density of middle thigh (HU)	0.017 (−0.017, 0.052)	0.318	4.259 (−7.859, 16.377)	0.486
L2 Trunk muscle area (cm^2^)	0.006 (−0.040, 0.051)	0.802	−10.34 (−26.269, 5.589)	0.200
L2 Trunk muscle density (HU)	0.019 (−0.020, 0.058)	0.333	6.026 (−7.830, 19.881)	0.389
Grip strength (kg)	0.042 (0.008, 0.076)	0.018	−2.214 (−14.551, 10.123)	0.722
TUG (s)	−0.032 (−0.064, 0.001)	0.054	−6.840 (−18.650, 4.970)	0.253
**Females**				
G.MaxM area (cm^2^)	0.039 (0.019, 0.059)	<0.001	10.619 (1.015, 20.224)	0.030
G.MaxM density (HU)	0.020 (0.001, 0.040)	0.043	3.319 (−6.179, 12.817)	0.491
Muscle area of middle thigh (cm^2^)	0.035 (0.014, 0.057)	0.002	3.121 (−7.109, 13.351)	0.548
Muscle density of middle thigh (HU)	0.025 (0.006, 0.044)	0.009	6.620 (−3.250, 16.490)	0.187
L2 Trunk muscle area (cm^2^)	0.010 (−0.013, 0.033)	0.384	1.372 (−9.153, 11.897)	0.797
L2 Trunk muscle density (HU)	−0.011 (−0.033, 0.011)	0.306	−4.450 (−14.628, 5.728)	0.389
Grip strength(kg)	0.014 (−0.004, 0.033)	0.127	−3.435 (−12.211, 5.340)	0.441
TUG (s)	−0.010 (−0.029, 0.009)	0.292	−2.006 (−10.968, 6.955)	0.659

*SD, standard deviance; BMD, bone mineral density; TH, total hip; aBMD, areal bone mineral density; vBMD, volumetric bone mineral density; CI, confidence interval; G.MaxM, gluteus maximus muscle; TUG, Timed Up and Go test*.

a *Adjusted for age, body mass index (BMI), EuroQol five-dimension score (EQ-5D)*.

b *β for standard deviance increase of continuous muscle variables*.

### Femoral Neck BMD

Adjusted β and 95% Cl of four femoral neck BMD sites (FN aBMD, Neck Int. vBMD, Neck Trab. vBMD, and Neck Cort. vBMD) with continuous muscle indexes per sex-specific SD increase are shown in Table 3. No significance was found for Neck Int. vBMD and Neck Cort. vBMD (all *P* > 0.05). In women, 13.965 and 9.402 mg/cm^3^ of Neck Trab. vBMD increased with one SD increase of G.MaxM area (95% CI, 5.676–22.254; *P* = 0.001) and muscle area of middle thigh (95% CI, 0.519–18.285; *P* = 0.038), while this significance was not shown in men (both *P* > 0.05). FN aBMD was associated with G.MaxM area in women (β, 0.034; 95% CI, 0.015–0.052; *P* = 0.001), G.MaxM density in men (β, 0.030; 95% CI, 0.001–0.060; *P* = 0.043), muscle area of middle thigh in women (β, 0.028; 95% CI, 0.008–0.048; *P* = 0.006), and muscle density of middle thigh in women (β, 0.020; 95% CI, 0.003–0.038; *P* = 0.022).

**Table 3:** Adjusted β and 95% CIs for sex-specific SD increase of various muscle indexes with femoral neck BMD^a,b^.

**Variables**	**FN aBMD (g/cm**^****2****^**)**		**Neck Int.vBMD (mg/cm**^****3****^**)**		**Neck Trab.vBMD (mg/cm**^****3****^**)**		**Neck Cort.vBMD (mg/cm**^****3****^**)**	
	**β (95% CI)**	***P*-value**	**β (95% CI)**	***P*-value**	**β (95% CI)**	***P*-value**	**β (95% CI)**	***P*-value**
**Males**								
G.MaxM area (cm^2^)	0.019 (−0.013, 0.050)	0.241	4.663 (−11.046, 20.371)	0.557	4.018 (−7.465, 15.501)	0.488	9.544 (−11.543, 30.631)	0.371
G.MaxM density (HU)	0.030 (0.001, 0.060)	0.043	10.027 (−4.406, 24.459)	0.171	6.956 (−3.615, 17.528)	0.194	18.933 (−0.275, 38.141)	0.053
Muscle area of middle thigh (cm^2^)	0.025 (−0.005, 0.055)	0.107	4.307 (−10.851, 19.466)	0.573	6.560 (−4.458, 17.578)	0.240	−6.238 (−26.637, 14.162)	0.545
Muscle density of middle thigh (HU)	0.023 (−0.006, 0.053)	0.121	8.869 (−6.083, 23.822)	0.241	6.350 (−4.593, 17.294)	0.252	8.461 (−11.752, 28.674)	0.407
L2 Trunk muscle area (cm^2^)	−0.006 (−0.045, 0.033)	0.770	−12.336 (−31.908, 7.237)	0.213	−5.015 (−19.564, 9.534)	0.494	−17.001 (−42.478, 8.476)	0.188
L2 Trunk muscle density (HU)	0.023 (−0.010, 0.057)	0.164	12.940 (−3.887, 29.768)	0.130	11.030 (−1.313, 23.374)	0.079	9.913 (−12.255, 32.082)	0.376
Grip strength (kg)	0.023 (−0.008, 0.053)	0.139	−4.891 (−20.171, 10.389)	0.526	0.717 (−10.488, 11.923)	0.899	−11.510 (−31.974, 8.954)	0.267
TUG (s)	−0.027 (−0.055, 0.0004)	0.053	−7.318 (−22.001, 7.364)	0.324	−6.915 (−17.614, 3.783)	0.202	−6.815 (−26.642, 13.011)	0.496
**Females**								
G.MaxM area (cm^2^)	0.034 (0.015, 0.052)	0.001	10.857 (−0.677, 22.390)	0.065	13.965 (5.676, 22.254)	0.001	9.509 (−9.168, 28.185)	0.316
G.MaxM density (HU)	0.007 (−0.011, 0.026)	0.440	−1.650 (−13.026, 9.726)	0.775	−1.255 (−9.609, 7.099)	0.767	0.981 (−17.313, 19.274)	0.916
Muscle area of middle thigh (cm^2^)	0.028 (0.008, 0.048)	0.006	3.860 (−8.377, 16.097)	0.534	9.402 (0.519, 18.285)	0.038	2.600 (−17.093, 22.294)	0.795
Muscle density of middle thigh (HU)	0.020 (0.003, 0.038)	0.022	5.726 (−6.110, 17.562)	0.341	4.868 (−3.817, 13.552)	0.270	4.107 (−14.965, 23.178)	0.671
L2 Trunk muscle area (cm^2^)	0.012 (−0.010, 0.034)	0.274	−1.098 (−13.987, 11.792)	0.867	1.254 (−8.123, 10.632)	0.792	−0.282 (−20.570, 20.007)	0.978
L2 Trunk muscle density (HU)	−0.006 (−0.027, 0.014)	0.550	−7.124 (−19.564, 5.316)	0.260	−6.707 (−15.732, 2.318)	0.144	−8.640 (−28.254, 10.974)	0.385
Grip strength(kg)	0.013 (−0.005, 0.030)	0.151	−4.685 (−15.178, 5.808)	0.379	0.025 (−7.698, 7.748)	0.995	−3.572 (−20.472, 13.328)	0.677
TUG (s)	−0.004 (−0.022, 0.014)	0.636	0.100 (−10.627, 10.827)	0.985	−1.278 (−9.153, 6.597)	0.749	2.149 (−15.095, 19.393)	0.806

*SD, standard deviance; BMD, bone mineral density; FN, femoral neck; Int., integral; Trab., trabecular; Cort., cortical; aBMD, areal bone mineral density; vBMD, volumetric bone mineral density; CI, confidence interval; G.MaxM, gluteus maximus muscle; TUG, Timed Up and Go test*.

a *Adjusted for age, body mass index (BMI), EuroQol five-dimension score (EQ-5D)*.

b *β for standard deviance increase of continuous muscle variables*.

### Trochanter BMD

The adjusted results of general linear models for the associations between trochanter (TR) BMD and muscle indexes are presented in Table 4. More muscle indexes were observed to be related to TR aBMD, including G.MaxM area (men: *P* = 0.022; women: *P* < 0.001), G.MaxM density (men: *P* = 0.034; women: *P* = 0.002), muscle area of middle thigh (men: *P* = 0.046; women: *P* < 0.001), muscle density of middle thigh (only women: *P* = 0.002), and HGS (only men: *P* = 0.019). G.MaxM area was associated with TR Int. vBMD (β, 9.672; 95% CI, 2.430–16.914; *P* = 0.009) and TR Trab. vBMD (β, 6.342; 95% CI, 1.129–11.555; *P* = 0.017) in women, but not in men (*P* > 0.05). Associations with TR Cort. vBMD were found for G.MaxM area in men (β, 19.898; 95% CI, 0.924–38.871; *P* = 0.040) and in women (β, 15.426; 95% CI, 0.893–29.958; *P* = 0.038). Muscle density of the middle thigh in women was related significantly to TR Int.vBMD (β, 8.319; 95% CI, 0.898–15.740; *P* = 0.028) and TR Cort.vBMD (β, 17.294; 95% CI, 2.531–32.057; *P* = 0.022).

**Table 4:** Adjusted β and 95% CIs for sex-specific SD increase of various muscle indexes with trochanter BMD^a,b^.

**Variables**	**TR aBMD (g/cm**^****2****^**)**		**TR Int.vBMD (mg/cm**^****3****^**)**		**TR Trab.vBMD (mg/cm**^****3****^**)**		**TR Cort.vBMD (mg/cm**^****3****^**)**	
	**β (95% CI)**	***P*-value**	**β (95% CI)**	***P*-value**	**β (95% CI)**	***P*-value**	**β (95% CI)**	***P*-value**
**Males**								
G.MaxM area (cm^2^)	0.038 (0.006, 0.071)	0.022	8.835 (−2.620, 20.290)	0.129	4.226 (−3.858, 12.311)	0.301	19.898 (0.924, 38.871)	0.040
G.MaxM density (HU)	0.034 (0.003, 0.065)	0.034	5.328 (−5.384, 16.040)	0.325	4.037 (−3.458, 11.532)	0.287	15.473 (−2.261, 33.208)	0.086
Muscle area of middle thigh (cm^2^)	0.033 (0.001, 0.065)	0.046	4.408 (−6.760, 15.576)	0.435	5.315 (−2.450, 13.079)	0.177	5.189 (−13.562, 23.940)	0.584
Muscle density of middle thigh (HU)	0.008 (−0.024, 0.040)	0.628	2.988 (−8.122, 14.098)	0.594	4.590 (−3.139, 12.320)	0.241	3.338 (−15.298, 21.974)	0.723
L2 Trunk muscle area (cm^2^)	0.005 (−0.037, 0.048)	0.805	−5.723 (−20.163, 8.717)	0.432	−3.729 (−13.853, 6.396)	0.465	−1.420 (−24.973, 22.133)	0.905
L2 Trunk muscle density (HU)	0.008 (−0.028, 0.044)	0.654	4.450 (−8.040, 16.941)	0.480	5.300 (−3.394, 13.993)	0.228	6.264 (−14.040, 26.569)	0.540
Grip strength (kg)	0.038 (0.006, 0.070)	0.019	3.373 (−7.908, 14.654)	0.554	1.761 (−6.148, 9.671)	0.659	7.617 (−11.255, 26.489)	0.424
TUG (s)	−0.022 (−0.052, 0.008)	0.147	−4.490 (−15.347, 6.367)	0.413	−5.526 (−13.065, 2.012)	0.149	−4.144 (−22.390, 14.102)	0.653
**Females**								
G.MaxM area (cm^2^)	0.033 (0.018, 0.049)	<0.001	9.672 (2.430, 16.914)	0.009	6.342 (1.129, 11.555)	0.017	15.426 (0.893, 29.958)	0.038
G.MaxM density (HU)	0.024 (0.009, 0.039)	0.002	5.476 (−1.693, 12.645)	0.134	2.706 (−2.455, 7.867)	0.302	11.475 (−2.795, 25.745)	0.114
Muscle area of middle thigh (cm^2^)	0.030 (0.014, 0.047)	<0.001	5.124 (−2.608, 12.855)	0.193	5.005 (−0.517, 10.528)	0.075	9.299 (−6.115, 24.713)	0.235
Muscle density of middle thigh (HU)	0.023 (0.009, 0.037)	0.002	8.319 (0.898, 15.740)	0.028	2.980 (−2.401, 8.361)	0.276	17.294 (2.531, 32.057)	0.022
L2 Trunk muscle area (cm^2^)	0.012 (−0.006, 0.030)	0.194	1.181 (−6.759, 9.121)	0.769	−0.524 (−6.290, 5.243)	0.858	7.176 (−8.006, 22.358)	0.352
L2 Trunk muscle density (HU)	−0.009 (−0.026, 0.008)	0.290	−3.037 (−10.719, 4.645)	0.436	−2.655 (−8.228, 2.918)	0.348	−3.111 (−17.862, 11.639)	0.677
Grip strength(kg)	0.013 (−0.002, 0.027)	0.083	−1.006 (−7.675, 5.663)	0.766	−0.182 (−4.967, 4.603)	0.940	2.080 (−11.204, 15.363)	0.758
TUG (s)	−0.008 (−0.023, 0.006)	0.264	−0.696 (−7.498, 6.107)	0.840	−0.064 (−4.945, 4.817)	0.979	−0.386 (−13.939, 13.167)	0.955

*SD, standard deviance; BMD, bone mineral density; TR, trochanter; Int., integral; Trab., trabecular; Cort., cortical; aBMD, areal bone mineral density; vBMD, volumetric bone mineral density; CI, confidence interval; G.MaxM, gluteus maximus muscle; TUG, Timed Up and Go test*.

a *Adjusted for age, body mass index (BMI), EuroQol five-dimension score (EQ-5D)*.

b *β for standard deviance increase of continuous muscle variables*.

### Intertrochanter BMD

Table 5 describes the results from multivariate general linear models, assessing the associations of intertrochanter BMD with eight muscle indexes, including G. MaxM area and density, muscle area and density of the middle thigh, L2 Trunk muscle area and density, grip strength, and TUG. Compared to vBMD, IT aBMD showed statistical significance with G.Max area (men: *P* = 0.028; women: *P* = 0.001), G.MaxM density in men (*P* = 0.010), muscle area of middle thigh in women (*P* = 0.001), muscle density of middle thigh in women (*P* = 0.023), and grip strength in men (*P* = 0.016). IT Trab. vBMD in women was associated with G.MaxM area (β, 9.865; 95% CI, 2.611–17.119; *P* = 0.008), and muscle area of middle thigh (β, 7.759; 95% CI, 0.060–15.458; *P* = 0.048).

**Table 5:** Adjusted β and 95% CIs for sex-specific SD increase of various muscle indexes with intertrochanter BMD^a,b^.

**Variables**	**IT aBMD (g/cm**^****2****^**)**		**IT Int.vBMD (mg/cm**^****3****^**)**		**IT Trab.vBMD (mg/cm**^****3****^**)**		**IT Cort.vBMD (mg/cm**^****3****^**)**	
	**β (95% CI)**	***P*-value**	**β (95% CI)**	***P*-value**	**β (95% CI)**	***P*-value**	**β (95% CI)**	***P*-value**
**Males**								
G.MaxM area (cm^2^)	0.046 (0.005, 0.087)	0.028	3.313 (−10.635, 17.261)	0.638	7.367 (−2.207, 16.940)	0.130	13.120 (−13.110, 39.350)	0.323
G.MaxM density (HU)	0.051 (0.012, 0.090)	0.010	7.557 (−5.290, 20.403)	0.245	7.546 (−1.305, 16.398)	0.094	16.542 (−7.660, 40.744)	0.178
Muscle area of middle thigh (cm^2^)	0.027 (−0.014, 0.068)	0.196	−7.624 (−20.995, 5.747)	0.260	2.339 (−7.014, 11.693)	0.620	−20.852 (−45.897, 4.192)	0.102
Muscle density of middle thigh (HU)	0.025 (−0.015, 0.065)	0.214	2.391 (−10.977, 15.759)	0.723	7.320 (−1.841, 16.480)	0.116	−5.862 (−31.106, 19.381)	0.645
L2 Trunk muscle area (cm^2^)	0.008 (−0.044, 0.061)	0.753	−13.316 (−30.929, 4.297)	0.136	−5.243 (−17.423, 6.938)	0.394	−12.537 (−45.449, 20.376)	0.450
L2 Trunk muscle density (HU)	0.027 (−0.018, 0.071)	0.240	5.099 (−10.319, 20.516)	0.512	8.482 (−1.908, 18.871)	0.108	4.590 (−23.950, 33.131)	0.749
Grip strength (kg)	0.049 (0.009, 0.090)	0.016	−7.366 (−20.860, 6.128)	0.281	1.569 (−7.872, 11.011)	0.742	−16.418 (−41.840, 9.005)	0.203
TUG (s)	−0.036 (−0.073, 0.001)	0.059	−8.238 (−21.217, 4.742)	0.210	−7.026 (−16.005, 1.952)	0.123	0.382 (−24.377, 25.141)	0.976
**Females**								
G.MaxM area (cm^2^)	0.046 (0.020, 0.071)	0.001	10.085 (−1.714, 21.883)	0.093	9.865 (2.611, 17.119)	0.008	9.435 (−14.915, 33.785)	0.445
G.MaxM density (HU)	0.018 (−0.007, 0.043)	0.149	3.482 (−8.126, 15.090)	0.555	1.662 (−5.567, 8.892)	0.651	8.707 (−15.079, 32.492)	0.471
Muscle area of middle thigh (cm^2^)	0.045 (0.019, 0.072)	0.001	0.538 (−11.972, 13.049)	0.932	7.759 (0.060, 15.458)	0.048	−5.789 (−31.423, 19.845)	0.656
Muscle density of middle thigh (HU)	0.027 (0.004, 0.051)	0.023	6.089 (−5.996, 18.174)	0.321	5.992 (−1.498, 13.482)	0.116	2.750 (−22.095, 27.594)	0.827
L2 Trunk muscle area (cm^2^)	0.012 (−0.017, 0.041)	0.402	2.615 (−10.196, 15.425)	0.687	1.782 (−6.280, 9.843)	0.663	2.376 (−23.273, 28.024)	0.855
L2 Trunk muscle density (HU)	−0.015 (−0.042, 0.012)	0.282	−4.142 (−16.547, 8.264)	0.510	−3.798 (−11.592, 3.996)	0.337	−10.152 (−34.959, 14.656)	0.420
Grip strength(kg)	0.015 (−0.008, 0.038)	0.206	−5.494 (−16.201, 5.213)	0.313	−1.878 (−8.558, 4.802)	0.580	−9.182 (−31.156, 12.792)	0.411
TUG (s)	−0.011 (−0.035, 0.013)	0.377	−5.722 (−16.642, 5.198)	0.302	−3.415 (−10.215, 3.385)	0.323	−1.145 (−23.602, 21.313)	0.920

*SD, standard deviance; BMD, bone mineral density; IT, intertrochanter; Int., integral; Trab., trabecular; Cort., cortical; aBMD, areal bone mineral density; vBMD, volumetric bone mineral density; CI, confidence interval; G.MaxM, gluteus maximus muscle; TUG, Timed Up and Go test*.

a *Adjusted for age, body mass index (BMI), EuroQol five-dimension score (EQ-5D)*.

b *β for standard deviance increase of continuous muscle variables*.

### Cortical Thickness

The adjusted association of cortical thickness from total hip, femoral neck, trochanter, intertrochanter sites were evaluated with eight muscle index, including G.MaxM area and density, muscle area and density of middle thigh, L2 Trunk muscle area and density, grip strength, and TUG, which were presented in Table 6. Only male TUG was found to be associated with TH CorThick (β, 0.075; 95% CI, 0.004–0.147; *P* = 0.040), FN CortThick (β, 0.070; 95% CI, 0.002–0.138; *P* = 0.044), TR CortThick (β, 0.090; 95% CI, 0.010–0.170; *P* = 0.027), but no significance was found in other indexes.

**Table 6:** Adjusted β and 95% CIs for sex-specific SD increase of various muscle indexes with cortical thickness^a,b^.

**Variables**	**TH CortThick (mm)**		**Neck CortThick (mm)**		**TR CortThick (mm)**		**IT CortThick (mm)**	
	**β (95% CI)**	***P*-value**	**β (95% CI)**	***P*-value**	**β (95% CI)**	***P*-value**	**β (95% CI)**	***P*-value**
**Males**								
G.MaxM area (cm^2^)	0.038 (−0.039, 0.115)	0.334	0.047 (−0.026, 0.120)	0.206	0.046 (−0.041, 0.132)	0.295	0.022 (−0.070, 0.114)	0.635
G.MaxM density (HU)	−0.027 (−0.099, 0.045)	0.462	−0.007 (−0.076, 0.062)	0.841	−0.031 (−0.112, 0.050)	0.446	−0.043 (−0.129, 0.042)	0.318
Muscle area of middle thigh (cm^2^)	0.027 (−0.048, 0.102)	0.469	0.038 (−0.033, 0.109)	0.287	0.045 (−0.039, 0.128)	0.292	0.004 (−0.085, 0.094)	0.923
Muscle density of middle thigh (HU)	0.002 (−0.073, 0.076)	0.960	0.027 (−0.044, 0.097)	0.454	0.001 (−0.082, 0.084)	0.979	−0.019 (−0.108, 0.070)	0.669
L2 Trunk muscle area (cm^2^)	0.011 (−0.080, 0.102)	0.813	0.011 (−0.070, 0.092)	0.784	0.019 (−0.083, 0.121)	0.707	0.007 (−0.107, 0.121)	0.900
L2 Trunk muscle density (HU)	−0.001 (−0.079, 0.077)	0.974	0.015 (−0.055, 0.085)	0.671	0.012 (−0.075, 0.100)	0.780	−0.023 (−0.121, 0.075)	0.643
Grip strength (kg)	0.027 (−0.049, 0.103)	0.477	0.040 (−0.032, 0.112)	0.274	0.045 (−0.040, 0.130)	0.292	−0.009 (−0.099, 0.082)	0.851
TUG (s)	0.075 (0.004, 0.147)	0.040	0.070 (0.002, 0.138)	0.044	0.090 (0.010, 0.170)	0.027	0.082 (−0.003, 0.168)	0.059
**Females**								
G.MaxM area (cm^2^)	0.019 (−0.028, 0.065)	0.431	0.017 (−0.028, 0.061)	0.459	0.019 (−0.031, 0.069)	0.445	0.011 (−0.050, 0.073)	0.717
G.MaxM density (HU)	−0.032 (−0.077, 0.013)	0.160	−0.033 (−0.076, 0.010)	0.135	−0.018 (−0.066, 0.031)	0.475	−0.053 (−0.112, 0.006)	0.080
Muscle area of middle thigh (cm^2^)	−0.009 (−0.058, 0.040)	0.723	−0.002 (−0.049, 0.045)	0.940	−0.004 (−0.056, 0.049)	0.890	−0.018 (−0.083, 0.046)	0.580
Muscle density of middle thigh (HU)	−0.020 (−0.067, 0.027)	0.398	−0.017 (−0.062, 0.028)	0.460	−0.018 (−0.068, 0.033)	0.486	−0.022 (−0.084, 0.040)	0.479
L2 Trunk muscle area (cm^2^)	0.027 (−0.021, 0.076)	0.268	0.017 (−0.031, 0.066)	0.484	0.025 (−0.026, 0.075)	0.335	0.039 (−0.027, 0.105)	0.239
L2 Trunk muscle density (HU)	−0.003 (−0.050, 0.045)	0.908	−0.008 (−0.055, 0.039)	0.742	−0.000 (−0.049, 0.049)	1.000	0.003 (−0.061, 0.067)	0.919
Grip strength(kg)	0.001 (−0.041, 0.043)	0.973	−0.020 (−0.060, 0.020)	0.333	0.008 (−0.037, 0.053)	0.737	0.009 (−0.046, 0.065)	0.747
TUG (s)	−0.022 (−0.065, 0.020)	0.302	−0.005 (−0.046, 0.036)	0.820	−0.013 (−0.058, 0.033)	0.586	−0.047 (−0.103, 0.009)	0.098

*SD, standard deviance; BMD, bone mineral density; CortThick, cortical thickness; aBMD, areal bone mineral density; vBMD, volumetric bone mineral density; CI, confidence interval; G.MaxM, gluteus maximus muscle; TUG, Timed Up and Go test*.

a *Adjusted for age, body mass index (BMI), EuroQol five-dimension score (EQ-5D)*.

b *β for standard deviance increase of continuous muscle variables*.

## Discussion

The novel finding of this study was that gluteus maximus muscle cross-sectional area but not density was associated with trochanter cortical vBMD suggesting that muscle size is at this region, is more important than muscle density to localized bone. This observation indicates the potential value of this muscle being a good target to improve the bone strength via for example appropriate physical exercise (23). We also found the site-specific effects in the associations of muscle and bone density, namely trunk muscle density, and size were not associated with proximal femur bone density. Notably, the association of hip/thigh muscle with femoral neck bone was weaker than those with trochanter and intertrochanter ROIs. Furthermore, compared to muscle density, muscle size showed better associations with vBMD.

Previous studies have found that the thickness of soft tissue near the greater trochanter or thigh are protective of hip fracture (24–26). These reports provided evidence that soft tissue thickness may influence hip fracture risk by attenuating external forces applied to the femur during a sideways fall. Our results show that the increase of G.MaxM area is associated with higher TR cortical vBMD in both men and women after adjustment for covariates, indicating that larger gluteus muscle size translates to higher cortical bone strength. Our data also imply that the thickness of soft tissue associated with hip fracture risk might not be limited to attenuating external forces, but also by strengthening the neighboring cortical bone. It is well-known that most of the compressive and bending strength of a long bone is in its cortical shell (27, 28). The gluteus muscle is considered as one of the strongest muscles in the body and inserted on the gluteal tuberosity of the femur (G.MaxM) and on the greater trochanter of the femur (gluteus medius/minimus muscle). The anatomy relation could partly explain the association of the G.MaxM area and TR cortical vBMD as the gluteus muscle mechanical loading and physical forces created by gluteus muscle contractions would directly affect the adjacent trochanter cortex. Furthermore, in women the muscle and bone interaction effect was more dominant than that in men as G.MaxM area also correlated with TH and TR Int. vBMD and Neck, TR and IT Trab. vBMD in women but not in men. This could partly explain the sex-related differences in predictive value of hip fracture risk in previous studies. One women cohort study found each standard deviation decrease in trochanteric soft tissue thickness increased fracture risk by 80% (24). However, in the MrOS Study, no association was found between hip fracture and trochanteric soft tissue thickness (25). The close ties of gluteus muscle and the proximal femur bone especially in women indicate this muscle could be a good target to improve the bone strength. In addition, the gluteus muscle plays an important role on gait stability, which is crucial for the reduction of hip fracture risk.

Bone and muscle experience organogenesis through tightly orchestrated gene activation and inactivation programs to ensure that bone and muscle develop synchronously (29), which results generally in larger bones developing together with larger muscles. It is well-recognized that aBMD measures the superposition of cortical and trabecular bone and results are dependent on bone size due to the two-dimensional projection nature. Therefore, the interpretation of the relationship between muscle and aBMD should be done with caution and take into account for bone size. However, our data demonstrated that, compared to vBMD, aBMD of the ROIs at the proximal femur was better associated with muscle variables in both sexes. Therefore, the influence of bone size on vBMD and aBMD in the correlation with muscle parameters needs further study.

Muscle density is a good indicator to quantify the lipid infiltration of skeletal muscle which appears to contribute to age-related decline in skeletal muscle function (30). However, in our study, the muscle density was not associated with vBMD of most ROIs in both sexes except for mid-thigh muscle density being associated with TR integral and cortical vBMD. We postulate that the muscle forces generated in the thigh and through the hip flexors and extensors are very likely to be great. As such the overall muscle size of hip/thigh will increase the force to the bone and would suggest that mechanical forces are significant to bone at this site. Further study is warranted to confirm this possibility.

The associations of physical performance (HG and TUG) and bone parameters were poor in this study. Hand grip strength was only associated with TR aBMD and IT aBMD in men after adjustment. The finding of no associations of hand grip strength and vBMD at the femur is consistent with the results of a recent MrOS study (13). However, Chalhoub et al. found an association between grip strength and the geometry and strength parameters at the radius, but not at the tibia, suggesting site-specific effects. We can also observe the similar site-specific effects in our data, namely no associations of trunk muscle density and size with the proximal femur bone density. TUG was not associated with BMD in this study. The TUG test includes walking, turning and the performance of sitting to standing, and provides a comprehensive representation of the balance and functional mobility capacities of an individual. The nature of TUG in reflecting multiple complex pathoetiologies may introduce the complexity in exploring such associations.

There are several limitations in this study. Firstly, the cross-sectional design does not enable the exploration of how changes in muscle quality and size might affect BMD. Secondly, our study population was drawn from community-dwelling Chinese adults which could limit the generalizability of the results to other ethnicities. Thirdly, our cohort comprised of adults aged 59–85 years and therefore data are probably not generalizable to young and middle-age adults, and the smaller numbers of men compared to women in the study may explain some of the sex-specific associations. Fourthly, the lack of data on physical activity may moderate the interpretation of this study outcomes.

In conclusion, we observed positive associations of the gluteus and thigh muscle size with proximal femur volumetric BMD. Specifically, gluteus maximus muscle CSA was associated with trochanter cortical vBMD in both sexes. Therefore, hip and thigh muscle size may represent a more clinically meaningful target for osteoporosis treatment, as well as for hip fracture prevention.

## Data Availability Statement

The original contributions presented in the study are included in the article/supplementary material, further inquiries can be directed to the corresponding author/s.

## Ethics Statement

The studies involving human participants were reviewed and approved by Ethics Committee of Beijing Jishuitan Hospital. The patients/participants provided their written informed consent to participate in this study.

## Author Contributions

LY, ZX, and LW: manuscript drafting. LY and LW: statistical analyses. WS, MY, AY, and XW: site coordination. ZX, LW, YZ, YS, and YL: QCT measurements. LW, WL, XC, and KE: study designing and project coordination. LW, WL, GB, AV-V, XC, KH, and KE: manuscript revisions. All authors contributed to the article and approved the submitted version.

## Conflict of Interest

The authors declare that the research was conducted in the absence of any commercial or financial relationships that could be construed as a potential conflict of interest.
